# Connecting the Dots: Exploring the Association Between Systemic Lupus Erythematosus and Thyroid Disorders

**DOI:** 10.7759/cureus.74469

**Published:** 2024-11-26

**Authors:** Syed Muhammad Hayyan Nishat, Asma A Alzaabi, Fatema M Alzaabi, Dana J Al Tarawneh, Yusuf J Al Tarawneh, Abdallah Khan, Mohammed Abdul Muqsit Khan, Tabish W Siddiqui, Raqshan W Siddiqui, Shiza W Siddiqui

**Affiliations:** 1 Internal Medicine, Ras Al Khaimah (RAK) Medical and Health Sciences University, Ras Al Khaimah, ARE; 2 Research, Dubai Medical College, Dubai, ARE

**Keywords:** autoimmune disease, autoimmune thyroid disorder, hyperthyroidism, hypothyroidism, systemic lupus erythematosus, thyroid disorders

## Abstract

Systemic lupus erythematosus (SLE) is a complex autoimmune disease marked by chronic inflammation and tissue damage that impacts multiple organ systems and diminishes the quality of life. Among the frequent comorbidities in SLE, thyroid dysfunction, including hypothyroidism and hyperthyroidism, stands out due to its high prevalence and common autoimmune basis. This review examines the epidemiological, genetic, and immunological factors that link SLE with autoimmune thyroid diseases such as Hashimoto’s thyroiditis and Graves’ disease. These overlapping mechanisms suggest a shared pathophysiological foundation that increases the risk of thyroid dysfunction in SLE patients. Clinically, distinguishing thyroid dysfunction from SLE symptoms, such as fatigue and cognitive difficulties, remains challenging, making regular thyroid screening in SLE patients essential. A multidisciplinary approach, bringing together rheumatologists and endocrinologists, is crucial to provide comprehensive care and improve outcomes for patients managing both conditions.

## Introduction and background

Systemic lupus erythematosus (SLE) is a multifaceted autoimmune disease characterized by chronic inflammation and tissue damage affecting multiple organ systems, which can significantly impair quality of life and increase morbidity and mortality [1]. Globally, the prevalence of SLE varies, with higher rates reported in African, Hispanic, and Asian populations compared to Caucasians [2]. The disease predominantly affects women of childbearing age, contributing to substantial healthcare burdens and socioeconomic impacts [3].

A recognized comorbidity in patients with SLE is thyroid dysfunction, which includes both hypothyroidism and hyperthyroidism, often mediated by autoimmune mechanisms [4]. There is a considerably greater incidence of thyroid abnormalities in SLE patients than in the general population, suggesting a pathophysiologic link between the two conditions [5].

Hashimoto’s thyroiditis, an autoimmune thyroid condition that causes hypothyroidism, is prevalent among SLE patients and is defined by the presence of anti-thyroid peroxidase antibodies (AbTPOs) and lymphocytic infiltration of the thyroid gland [6]. Graves’ disease, an autoimmune thyroid disease (AITD) that causes hyperthyroidism, is characterized by the development of thyroid-stimulating immunoglobulins (TSI), which promote excessive thyroid hormone synthesis [7].

Shared genetic and immunological factors play important roles in the relationship between SLE and thyroid disorders. Both conditions exhibit common pathways involving immune system dysregulation, such as elevations in interferon-gamma (IFN-γ) and specific genetic polymorphisms [4,8]. Such an overlap indicates a common genetic basis and underlies the need for regular screening so that thyroid dysfunction is diagnosed early and managed effectively among patients of SLE [1].

In this article, we aim to discuss the relationship between SLE and thyroid diseases by analyzing cross-sectional studies, case-control studies, retrospective studies, and review articles. Our goal is to thoroughly explore common genetic factors, immunological mechanisms, and appropriate multidisciplinary management strategies.

## Review

Methodology

Search Strategy

**Figure 1:**
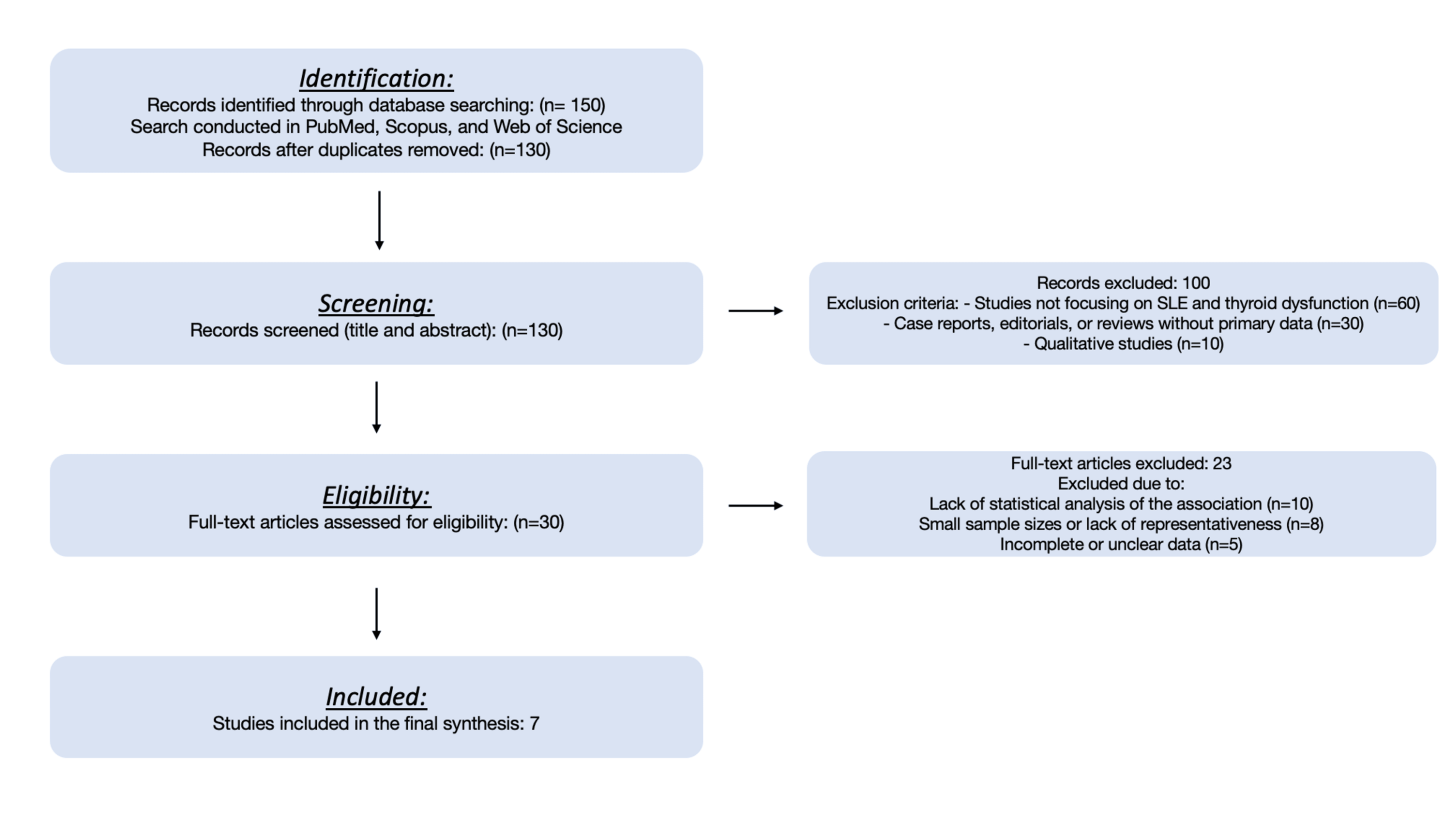
PRISMA flowchart of selected articles. PRISMA: Preferred Reporting Items for Systematic Reviews and Meta-Analyses; SLE: systemic lupus erythematosus

We conducted a systematic review following the Preferred Reporting Items for Systematic Reviews and Meta-Analyses (PRISMA) guidelines. A comprehensive search was performed in databases, including PubMed, Scopus, and Web of Science, using keywords such as “SLE and thyroid disorders,” “SLE and Hashimoto’s thyroiditis,” and “SLE and Graves’ disease.” Boolean operator (AND) was employed to refine search results. The search yielded 150 articles, of which 130 remained after duplicates were removed. Titles and abstracts of these articles were screened for relevance, and 30 full-text articles were further assessed for eligibility. After applying the inclusion and exclusion criteria, seven studies were included in the final synthesis. The study selection process is outlined in the PRISMA flowchart (Figure 1).

Inclusion and Exclusion Criteria

Inclusion criteria targeted observational studies (cross-sectional, cohort, and meta-analyses) that examined the relationship between SLE and thyroid dysfunction published between January 2000 and September 2024. Studies investigating the prevalence of hypothyroidism and hyperthyroidism in SLE patients, thyroid-specific antibodies, genetic overlaps, or immune mechanisms were included. Exclusion criteria followed PRISMA recommendations, excluding case reports, editorials, reviews without primary data (n = 30), and qualitative studies (n = 10). Full-text articles were excluded if they lacked statistical analyses (n = 10), had small sample sizes or lacked representativeness (n = 8), or presented incomplete or unclear data (n = 5).

Results

The initial search identified 150 articles. Following PRISMA-guided screening, 100 articles were excluded due to irrelevance (n = 60), being case reports or reviews without primary data (n = 30), or being qualitative studies (n = 10). After assessing 30 full-text articles, 23 were excluded for lack of statistical analysis (n = 10), small sample size or representativeness (n = 8), or incomplete data (n = 5). Finally, seven studies were included for review. The detailed process is summarized in the PRISMA flowchart (Figure 1).

Pathophysiology

Autoimmunity in Systemic Lupus Erythematosus

Genetic susceptibility: Variants in the human leukocyte antigens (HLA) *HLA-DR2* and *HLA-DR3* genes have been strongly associated with SLE, with specific haplotypes of these genes significantly contributing to autoantibody production and disease susceptibility, as revealed by Graham et al. [9]. This genetic predisposition highlights a broader risk for autoimmune diseases, including hypothyroidism, within this population [9]. These genes encode proteins responsible for presenting antigens to T cells, thereby influencing the immune response and underscoring their critical role in the pathogenesis of SLE [9]. Genes such as interferon regulatory factor 5 (*IRF5*), signal transducer and activator of transcription 4 (*STAT4*), and protein tyrosine phosphatase non-receptor type 22 (*PTPN22*) play critical roles in immune regulation and have been linked to SLE susceptibility [10].

SLE and AITDs exhibit significant genetic overlap, particularly within the major histocompatibility complex (MHC) region [11,12]. It has previously been reported that variants in MHC class II genes predispose to an increased risk of development of these autoimmune conditions [11,13]. For example, the *HLA-DRB103:01* and *HLA-DRB115:03* haplotypes have been linked to an increased risk of both SLE and thyroid disorders [14,15]. These genetic variants associated with susceptibility to autoimmune diseases provide insights into the mechanisms behind the loss of immune tolerance, affecting antigen presentation and T-cell responses, and contribute to the development of autoantibodies and clinical disease [16].

B-cell dysregulation: In SLE, nuclear antigens such as anti-double-stranded DNA (anti-dsDNA) and anti-Smith are produced by B cells [17]. These autoantibodies form immune complexes, which then deposit in tissues, leading to inflammation and damage [16]. Checkpoints that normally eliminate or inactivate self-reactive B cells fail in SLE, allowing these cells to survive and produce autoantibodies [18]. Key tolerance checkpoints include central tolerance in the bone marrow, where autoreactive B cells are either deleted or edited, and peripheral tolerance, where anergy, deletion, or regulation by T cells helps control autoreactive B cells [19]. Defects in these checkpoints contribute to the survival of autoreactive B cells and the production of pathogenic autoantibodies [19].

T-cell dysregulation: Both T helpers 1 and 17 (Th1 and Th17) cells are particularly active in SLE, producing cytokines such as IFN-γ and interleukin 17 (IL-17), which promote inflammation [20]. Th2 cells, while less prominent, also contribute by helping B cells produce autoantibodies [21]. Regulatory T cells (Tregs) that normally suppress immune responses are dysfunctional in SLE, failing to control autoreactive T and B cells [22].

Cytokine imbalance: Elevated levels of type I INFs (e.g., IFN-α) are a hallmark of SLE [23,24]. These cytokines activate immune cells, optimize antigen presentation, and stimulate autoantibody production [25]. Increased levels of IL-6, IL-10, and tumor necrosis factor-alpha (TNF-α) contribute to the inflammatory environment in SLE, promoting tissue damage and disease progression [26].

Autoimmunity in Thyroid Disorders

Hashimoto’s thyroiditis: Variants in *HLA-DR3* and *HLA-DR5* have been associated with Hashimoto’s thyroiditis [27]. Cytotoxic T-lymphocyte-associated protein 4 (*CTLA4*) is another gene implicated in this condition, as demonstrated by a study that used reverse transcription polymerase chain reaction to analyze genetic markers in patients with hypothyroidism [28]. This study found that a variant of the *CTLA4* gene was more common in patients with elevated levels of thyroid-specific antibodies, such as anti-thyroglobulin antibody (anti-TG) and AbTPO, which are indicators of autoimmune thyroid dysfunction [28]. AbTPO and anti-TG antibodies target thyroid antigens, leading to thyroid cell destruction [29]. Th1 and Th17 responses drive the production of pro-inflammatory cytokines, such as IL-17 and IFN-γ. These cytokines contribute to chronic thyroid inflammation and hypothyroidism [30].

Graves’ disease: *CTLA4* and *PTPN22* variants are linked to Graves’ disease [31]. Other associated genes also include thyroid-stimulating hormone receptor (TSHR) [31]. TSIs bind to and activate the TSHR, causing excessive thyroid hormone production and hyperthyroidism [7]. Th2 cells produce IL-4 and IL-10, which support the production of TSI and other autoantibodies, driving the hyperthyroid state [32].

Shared Mechanisms in Systemic Lupus Erythematosus and Thyroid Disorders

Interferon pathway: In SLE, plasmacytoid dendritic cells (pDCs) generate significant levels of type I INFs in response to immune complexes [33]. These INFs activate various immune cells and perpetuate the autoimmune response [25]. In autoimmune thyroiditis, type I INFs promote the presentation of thyroid antigens to T cells and the activation of autoreactive B cells, leading to autoantibody production and thyroid inflammation [34].

Genetic overlap: Shared variants in the HLA region, such as *HLA-DR3*, increase susceptibility to both SLE and thyroid disorders by affecting antigen presentation and immune regulation [9,27]. Variants in genes such as *PTPN22* and *CTLA4* are implicated in both SLE and thyroid disorders, influencing T-cell activation and tolerance [31,35].

Immune dysregulation: Immune system dysregulation is a central feature of both SLE and AITDs [36,37]. In SLE, the excessive production of autoantibodies and inflammatory cytokines, such as IFN-γ and IL-17, drives widespread inflammation and tissue damage, activating immune pathways that exacerbate thyroid dysfunction [30,38]. By stimulating immune cells and enhancing thyroid antigen presentation, these cytokines contribute to thyroid overstimulation, which may lead to hyperthyroid manifestations [30]. Maile et al. further elaborated on the role of thyroid epithelial cells in AITD, demonstrating that they can present endogenous thyroid antigens to T cells, potentially intensifying immune responses in SLE-related hyperthyroidism [13]. Similarly, AITDs, such as Hashimoto’s thyroiditis and Graves’ disease, involve the production of abTPOs and autoantibodies targeting thyroglobulin, driving thyroid gland inflammation and dysfunction [39].

Emerging therapies that target the type I interferon pathway in SLE may reduce autoimmune inflammation and potentially lower the risk of concurrent AITDs by mitigating IFN-driven immune activity [40].

Environmental triggers: Environmental factors play a crucial role in the pathogenesis of both SLE and AITDs [41,42]. Infections, stress, and certain medications can act as triggers, initiating or exacerbating the autoimmune response in genetically predisposed individuals [43].

**Figure 2:**
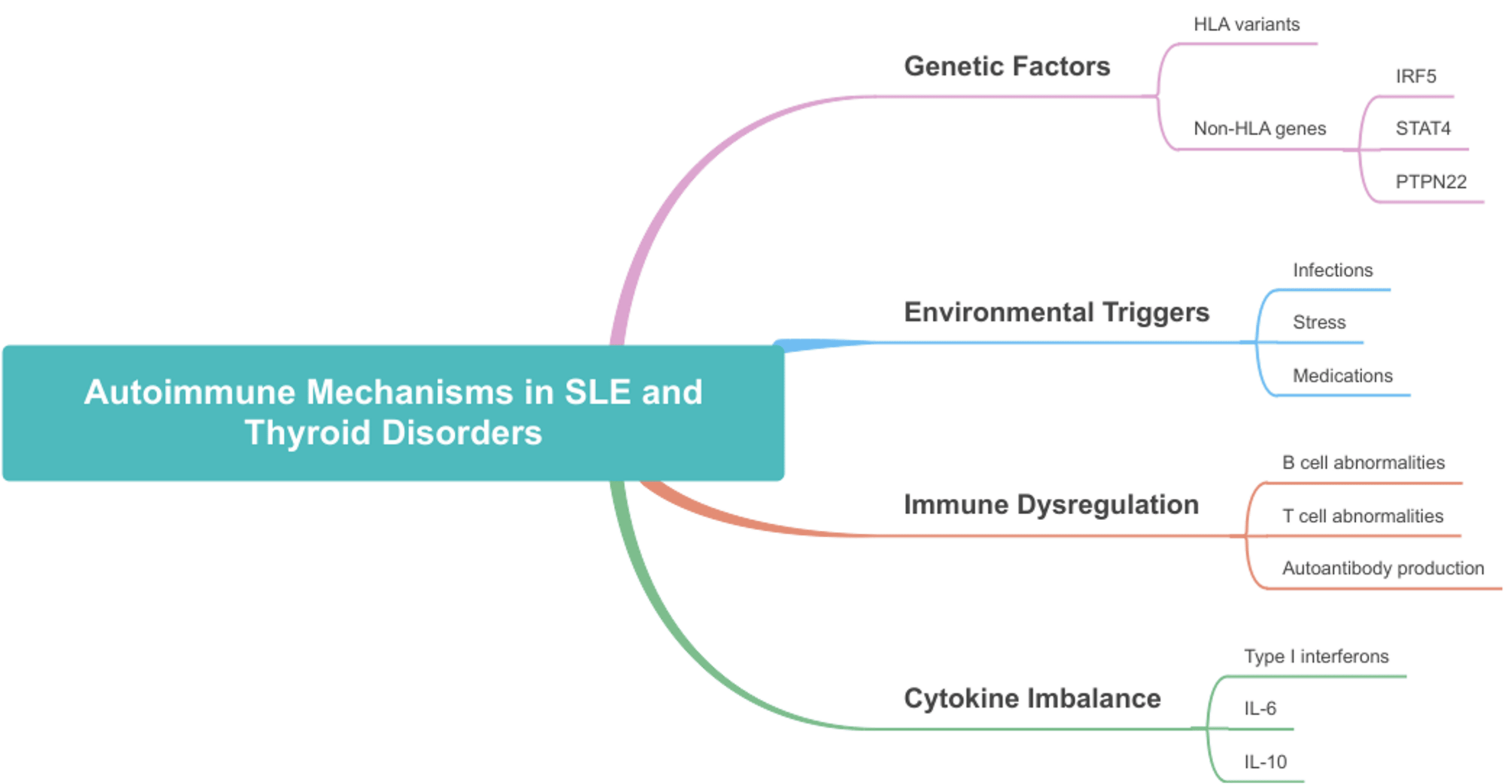
Overview of autoimmune mechanisms linking SLE and thyroid disorders. SLE: systemic lupus erythematosus; HLA: human leukocyte antigen; IRF5: interferon regulatory factor 5; STAT4: signal transducer and activator of transcription 4; PTPN22: protein tyrosine phosphatase, non-receptor type 22; IL-6: interleukin 6; IL-10: interleukin 10 Image credits: Syed Muhammad Hayyan Nishat.

Polyautoimmunity: The phenomenon of polyautoimmunity (PolyA), where patients with one autoimmune disease have an increased risk of developing another, is well-documented in the relationship between SLE and thyroid disorders [44]. For over 50 years, studies have shown a higher prevalence of thyroid autoimmunity in patients with SLE compared to the general population, with prevalence rates ranging from 18% to 32% [1,4]. The prevalence of AITD in SLE patients aligns with findings from Santos-Moreno et al., who reported that 34% of SLE patients exhibit PolyA, with AITD among the frequently co-occurring conditions [44]. Although the exact mechanism is unclear, the co-occurrence of these conditions may be influenced by overlapping immunological processes and genetic factors that predispose individuals to autoimmune diseases [42]. These overlapping mechanisms are visually summarized in Figure 2.

Epidemiology

Globally, SLE is a relatively rare condition, with prevalence estimates ranging from 20 to 150 cases per 100,000 individuals [45]. Higher rates are observed among African American, Hispanic, and Asian populations compared to Caucasians, with a striking female predominance, particularly among women of childbearing age [5,8]. In contrast, thyroid disorders are among the most common endocrine disorders worldwide, with hypothyroidism affecting about 1-2% of the general population and hyperthyroidism approximately 0.5-2% [46]. Both conditions share a similar gender disparity, with women being disproportionately affected [47,48].

The association between SLE and thyroid disorders has been a topic of interest for over five decades, with research consistently highlighting the higher prevalence of thyroid dysfunction among SLE patients compared to the general population [4]. It is essential to differentiate between thyroid dysfunction and thyroid autoimmunity in the context of SLE, as they represent distinct but related concepts.

Thyroid dysfunction encompasses a range of conditions characterized by abnormal thyroid hormone production or activity, including hypothyroidism, hyperthyroidism, and the presence of thyroid nodules. These disorders can result from multiple etiologies, including but not limited to autoimmune mechanisms. In contrast, thyroid autoimmunity specifically involves AITDs, such as Hashimoto’s thyroiditis or Graves’ disease, characterized by the presence of specific thyroid autoantibodies.

While many cases of thyroid dysfunction in SLE are attributable to autoimmune causes, not all are, underscoring the importance of distinguishing between these two entities in clinical practice.

Discussion

Hypothyroidism

Hypothyroidism is the most common thyroid disorder in SLE patients with studies reporting that primary hypothyroidism affects 15% to 19% of SLE patients, significantly higher than the 4.6% prevalence in the general population [4,49-52]. This increased prevalence is consistent across all age groups, with the highest rates in patients under 20 years of age and a higher likelihood in females [50,51].

A study conducted in Taiwan by Liu et al. found that hypothyroidism is significantly more prevalent in SLE patients compared to a matched control group [53]. The study reported that 8.5% of SLE patients developed hypothyroidism, a much higher incidence than the 2.2% observed in the control group, highlighting the strong association between SLE and thyroid dysfunction [53]. Similarly, a study in Brazil by Domingues et al. showed that 17.6% of SLE patients were affected by hypothyroidism compared to 5.5% in the control group with a notable increase in patients with lupus nephritis [54].

In Italy, Antonelli et al. found that hypothyroidism is significantly more common among SLE patients than in controls with increased serum TSH and AbTPO levels indicating a strong autoimmune component [49]. Shobha et al. conducted a descriptive cross-sectional study involving 100 SLE patients, reporting a remarkably high prevalence of hypothyroidism with clinical hypothyroidism observed in 60% of the patients and subclinical hypothyroidism in 24%​ [5]. This study highlighted that the prevalence of thyroid dysfunction was higher than previously reported in Indian populations and that thyroid dysfunction was more common among women and younger patients [5].

Further insights were provided by AL-Homood et al. in a retrospective study involving 151 SLE patients, identifying clinical hypothyroidism in 4.6% and subclinical hypothyroidism in 7.3% of the patients [54]. The study did not find a significant association between thyroid dysfunction and SLE disease activity, suggesting that hypothyroidism can occur independently of SLE exacerbations [54]. Elshair et al. conducted a cross-sectional study on 40 SLE patients revealing that 17.5% of the patients had subclinical hypothyroidism, and 7.5% had overt hypothyroidism, emphasizing the significant relationship between renal function and thyroid autoimmunity [55]. Watad et al. conducted a retrospective case-control study in Israel, involving 5,018 SLE patients and 25,090 matched controls, and reported that 15.58% of SLE patients had hypothyroidism compared to 5.75% in the control group​, highlighting a stronger association in males, younger patients, and across all socioeconomic statuses [50].

Hyperthyroidism

While less common than hypothyroidism, hyperthyroidism remains a notable thyroid dysfunction in SLE patients [4]. In Taiwan, Liu et al. identified hyperthyroidism in 6.4% of SLE patients, significantly higher than the general population, where the prevalence is approximately 0.5-2% [53]. Similar findings were observed by Domingues et al. in Brazil, with a 3.8% prevalence in SLE patients compared to 2.5% in controls [8]. These studies highlight the increased risk of hyperthyroidism in SLE patients across different regions.

Further insights into the association between subclinical hyperthyroidism and SLE were provided by AL-Homood et al. and Elshair et al. Both studies reported similar outcomes, with a prevalence of 3.3% and 5% of subclinical hyperthyroidism, respectively, in SLE patients [54,55]. These findings suggest a consistent pattern of thyroid dysfunction in SLE, with a potential link to renal function and autoimmune activity.

Prevalence of Thyroid Autoimmunity in Systemic Lupus Erythematosus

AITDs, such as Hashimoto’s thyroiditis and Graves’ disease, are prevalent in SLE patients, possibly reflecting shared autoimmune mechanisms and genetic predispositions [42]. The case-control study by Antonelli et al. revealed a significant prevalence of AITDs among SLE patients, with 71% of those with hypothyroidism testing positive for AbTPO [49]. This association was further supported by Shobha et al., who found that 25% of SLE patients had elevated AbTPO levels, indicating a high prevalence of AITD, particularly among women and younger patients [5].

Elshair et al. also contributed to this body of evidence, identifying that 33% of SLE patients had positive circulating AbTPO levels, emphasizing the strong link between thyroid autoimmunity and renal function in SLE [55]. Wei et al. added another dimension to this relationship by identifying serositis as a significant risk factor for AITD in SLE patients, while noting an inverse relationship between active SLE high anti-dsDNA titers/low complement component 3 (C3) levels and the risk of developing AITD [56]. These studies collectively underscore the intricate interplay between SLE and thyroid autoimmunity.

**Table 1:** Prevalence of thyroid dysfunction in SLE. SLE: systemic lupus erythematosus; AbTPO: anti-thyroid peroxidase antibodies; TSH: thyroid-stimulating hormone; T4: thyroxine; T3: triiodothyronine; AITD: autoimmune thyroid disease; ICD-9: International Classification of Diseases, Ninth Revision; ACR: American College of Rheumatology; LN: lupus nephritis; GFR: glomerular filtration rate; SELENA-SLEDAI: Safety of Estrogens in Lupus Erythematosus National Assessment-Systemic Lupus Erythematosus Disease Activity Index

Study/Reference, place of study	Study type	Sample size	Diagnostic criteria for SLE	Diagnostic criteria for thyroid disorders	Prevalence of hypothyroidism	Prevalence of hyperthyroidism	Prevalence of AITD	Risk factors/Subgroup analysis	Key findings
Shobha et al. [3], India	Descriptive cross-sectional study	100 SLE patients	Systemic Lupus International Collaborating Clinics 2012 lupus classification criteria	Thyroid function and AbTPO tests	Clinical hypothyroidism: 60%; subclinical hypothyroidism: 24%	Clinical hyperthyroidism: 0%; subclinical hyperthyroidism: 0%	25% (patients with elevated AbTPO)	Higher prevalence of thyroid dysfunction among women and younger patients	The prevalence of thyroid dysfunction was higher than in previous studies from India, with hypothyroidism being the most common abnormality. No significant impact on lupus activity was observed
Domingues et al. [8], Brazil	Case-control study	79 SLE patients, 159 controls	ACR 1997 criteria	Thyroid function tests and the presence of AITD	21.5% in SLE and 6.9% in controls	3.8% in SLE and 2.5% in controls	11.4 % in SLE and 13.8% in controls	Longer SLE duration was linked with thyroid dysfunction (p = 0.036); mild hypothyroidism was more frequent in SLE patients with anti-Smith antibodies (p = 0.029)	Hypothyroidism was more common in SLE patients, and AITD prevalence was high in both SLE and controls. Routine assessment of TSH and anti-thyroid antibodies is recommended for SLE patients
Antonelli et al. [49], Italy	Case-control study	213 SLE patientsand 426 controls	ACR 1997 criteria	Thyroid hormones, antithyroid antibodies, and thyroid ultrasonography	Clinical hypothyroidism: 6% more than control; subclinical hypothyroidism: 17% more than controls	N/A	AbTPO: present in 71% of SLE patients with hypothyroidism	Higher TSH and anti-TPO levels in female SLE patients; risk factors included female sex, positive AbTPO, hypoechoic pattern, and small thyroid	SLE patients, particularly women, had a significantly higher prevalence of hypothyroidism (both clinical and subclinical) and thyroid autoimmunity. A higher prevalence of Graves’ disease was also noted. The study emphasized the importance of routine thyroid function testing and ultrasonography in SLE patients
Watad et al. [50], Israel	Retrospective case-control study	5,018 SLE patients and 25,090 matched controls	Diagnosed based on physician documentation in medical records or hospital discharge summaries	Diagnosis extracted from the Clalit Health Services chronic diseases registry and validated by primary physicians	15.58% in SLE patients versus 5.75% in controls	N/A	The study confirmed that hypothyroidism was significantly more common in SLE patients, with a higher incidence of anti-thyroid antibodies. No specific figures were reported	Stronger association with hypothyroidism in males, younger patients, and across all socioeconomic statuses was noted	This study reported a significantly higher prevalence of hypothyroidism in SLE patients, particularly among males and younger individuals. The study underscored the need for regular thyroid screening in SLE patients due to the higher incidence of hypothyroidism and anti-thyroid antibodies
Liu et al. [53], Taiwan	Retrospective cohort	2,796	Catastrophic illness registration from the National Insurance Bureau and the ACR 1997 revised classification criteria for definite SLE	ICD-9 codes, confirmed by thyroid function test, autoantibodies, and medical and/or surgical intervention	8.5%	6.4%	5.4%	Higher relative risk of hypothyroidism and AITD in SLE patients with overlap syndrome; higher risk of severe complications (renal, CNS involvement)	SLE patients had a significantly higher rate of thyroid diseases compared to controls; SLE with thyroid diseases was linked to severe complications
AL-Homood et al. [54], Saudi Arabia	Retrospective study	151 SLE patients	Systemic Lupus International Collaborating Clinics classification criteria	Hypothyroidism: elevated TSH, low T4, and/or low T3, or treatment with thyroxine replacement therapy. Hyperthyroidism: low TSH, high T4/T3, or treatment with antithyroid medications	Clinical hypothyroidism: 4.6%; subclinical hypothyroidism: 7.3%	Clinical hyperthyroidism: 0.7%; subclinical hyperthyroidism: 3.3%	57% of hypothyroid patients positive for anti-Tg and AbTPO	No significant association was noted between thyroid dysfunction and SLE disease activity (SELENA-SLEDAI score)	Subclinical and overt hypothyroidism were common in patients with SLE, but no significant correlation was found with disease activity
Elshair et al. [55], Egypt	Cross-sectional study	20 patients with SLE without renal affection. 20 patients with LN 20 healthy controls	ACR criteria	Thyroid function tests and AbTPO tests	17.5% subclinical and 7.5% overt hypothyroidism	5% cases with subclinical hyperthyroidism	33% of patients had positive circulating AbTPO	A significant relationship was noted between renal function tests (urea, creatinine) and AbTPO; an inverse relationship was noted between estimated GFR and anti-TPO	High prevalence of thyroid dysfunction in SLE patients, especially those with LN; need for thyroid screening in SLE patients

Clinical Challenges and Management of Systemic Lupus Erythematosus With Thyroid Disorders

Differentiating between SLE and thyroid disorders presents significant diagnostic challenges, as thyroid dysfunction, particularly hypothyroidism, often mimics symptoms of SLE, such as fatigue, joint pain, and cognitive difficulties [5]. Accurate diagnosis relies on regular monitoring of thyroid function and AbTPO alongside SLE activity [5]. Given the high likelihood of thyroid dysfunction in SLE patients, proactive screening is essential to avoid misdiagnosis and ensure timely treatment [5].

Currently, there are no universally established guidelines for managing patients with both SLE and thyroid disorders. Clinicians, therefore, must rely on available literature and clinical experience, tailoring treatment to each patient’s unique presentation. This highlights the importance of a multidisciplinary approach, where endocrinologists, rheumatologists, and primary care providers collaborate closely to ensure comprehensive care. Routine screening for thyroid dysfunction is critical due to its higher prevalence in SLE patients [1]. Thyroid function tests and AbTPO assessments should be a part of the biochemical and immunological profiling for SLE patients [5]. Additionally, ultrasonography is recommended to detect structural thyroid abnormalities, such as nodules or diffuse enlargement [49]. High-risk patients, particularly women with positive AbTPO or small, hypoechoic thyroid glands, require more frequent follow-ups to manage potential thyroid dysfunction early [49].

Given the absence of specific guidelines, it may be advisable to approach treatment on an individual basis for each patient. Both SLE and thyroid disorders can significantly affect the quality of life, impacting physical and social well-being [57,58]. Patient-centered care involves tailoring treatments based on the patient’s unique clinical picture, disease activity, and personal circumstances [59]. Educating patients about their conditions, the importance of routine monitoring, and early detection of thyroid dysfunction is a vital part of care [59].

SLE patients with overlap syndromes were found to have a higher relative risk of thyroid diseases, such as hypothyroidism and AITD, complicating disease management [53]. Overlap syndromes increase the risk for renal and central nervous system involvement, making early recognition of thyroid disease in SLE patients essential [53]. Clinicians must stay vigilant in screening for thyroid dysfunction in patients with SLE or lupus nephritis [55].

The treatment of SLE commonly includes immunosuppressive drugs, corticosteroids, and antimalarials [60]. Corticosteroids, for instance, can suppress TSH secretion, potentially masking hypothyroidism or altering thyroid function tests [61,62]. Immunosuppressants such as azathioprine may also affect thyroid function, though the precise mechanisms of its interaction with thyroid tissue are still under investigation. Studies suggest that azathioprine could be effective in managing severe cases of Graves’ disease, raising the possibility of similar interactions when used in SLE treatment [63,64]. However, the clinical significance of these interactions remains unclear and warrants further research.

Limitations

While our study sheds light on the link between SLE and thyroid disorders, there are a few limitations worth noting. Some of the subgroups we reviewed had smaller sample sizes, which might introduce bias and make it harder to apply these findings to all SLE patients with thyroid dysfunction. As many studies we analyzed were cross-sectional, we cannot establish a cause-and-effect relationship, just an association. Additionally, differences in how thyroid dysfunction is diagnosed across studies could introduce bias, especially when comparing results for milder cases. Finally, the potential influence of medications commonly prescribed for SLE on thyroid function was not always accounted for, which may have impacted our findings.

Future directions

Larger, longer-term studies that can more clearly define the causal relationships between SLE and thyroid conditions should be the goal of future research to overcome these limitations. Research on immunological and genetic factors may help create tailored treatments by providing insights into the common pathophysiology. Furthermore, to enhance patient outcomes, consistent protocols for the screening and treatment of thyroid dysfunction, particularly in SLE patients, must be established. To improve complete care for people with both illnesses, interdisciplinary techniques should be investigated, such as cooperation between rheumatologists and endocrinologists.

## Conclusions

The association between SLE and thyroid disorders highlights a complex interplay of autoimmune processes that raises the risk of thyroid dysfunction, including both hypothyroidism and hyperthyroidism, in SLE patients. Recognizing this increased vulnerability, regular screening and proactive management are essential, particularly for women and younger individuals with SLE. Early detection and multidisciplinary care involving rheumatologists and endocrinologists can greatly improve patient outcomes, enabling tailored interventions that address the specific needs of SLE patients with thyroid issues. Establishing clear guidelines for managing these overlapping conditions can further support comprehensive care, ultimately improving the quality of life for individuals affected by SLE and thyroid dysfunction.
